# What Is the Optimal Geometry of Dissolving Microneedle Arrays? A Literature Review

**DOI:** 10.3390/pharmaceutics17010124

**Published:** 2025-01-17

**Authors:** Maira Visscher, Henderik W. Frijlink, Wouter L. J. Hinrichs

**Affiliations:** Department of Pharmaceutical Technology and Biopharmacy, University of Groningen, 9713 AV Groningen, The Netherlands; maira.visscher@rug.nl (M.V.); h.w.frijlink@rug.nl (H.W.F.)

**Keywords:** dissolving microneedles, geometry, design, drug delivery system, intradermal, transdermal, skin insertion, skin penetration, mechanical strength, optimization

## Abstract

The application of dissolving microneedle arrays (DMNAs) is an emerging trend in drug and vaccine delivery as an alternative for hypodermic needles or other less convenient drug administration methods. The major benefits include, amongst others, that no trained healthcare personnel is required and that the recipient experiences hardly any pain during administration. However, for a successful drug or vaccine delivery from the DMNA, the microneedles should be inserted intact into the skin. A successful penetration into the upper skin layers may be challenging because of the elastic nature of the skin; therefore, a minimum insertion force is required to overcome the total resistance force of the skin. In addition, the microneedles need to stay intact, which requires a certain mechanical strength, and be able to resist the required insertion force. In addition to the type of material with which the DMNAs are produced, the geometry of the DMNAs will also have a profound effect, not only on the mechanical strength but also on the number of insertions and penetration depth into the skin. In this review, the effects of shape, aspect ratio, length, width of the base, tip diameter and angle, and spacing of DMNAs on the aforementioned effect parameters were evaluated to answer the following question: ‘What is the optimal geometry of dissolving microneedle arrays?’.

## 1. Introduction

Currently, most biologicals such as vaccines and therapeutic proteins are administered by subcutaneous or intramuscular injection. These injections are accompanied by several disadvantages. First of all, storage and transport at refrigerated temperatures, the so-called cold chain, is often required to ensure the integrity of the drug. Next, trained health care personnel are required to prepare and administer the injection. Furthermore, sensory pain and needle phobia are associated with hypodermic needles. An alternative delivery technology to overcome these disadvantages is the use of dissolving microneedle arrays (DMNAs) instead of hypodermic needles. The possibilities and limitations of DMNAs have been described in various review articles [1,2]. The DMNA’s matrix is often prepared from (a combination of) water soluble polymers or saccharides. The release profile of the encapsulated drug depends on the nature of the matrix [3,4].

For a successful drug delivery from the DMNA into the skin, the DMNA needs to penetrate the stratum corneum. The stratum corneum is the top layer of the epidermis, with a thickness of 10–30 µm, and is most important for the protection against external influences [2,5,6]. To accomplish a successful penetration, the insertion force, which is applied onto the DMNA, needs to overcome the total resistance force of the skin, which consists of the stiffness, bending, buckling, cutting, and friction forces, as presented in Equation (1) [7].

(1) $$F_{insertion}=F_{stiffness}+F_{bending}+F_{buckling}+F_{cutting}+F_{friction}$$

**Figure 1:**
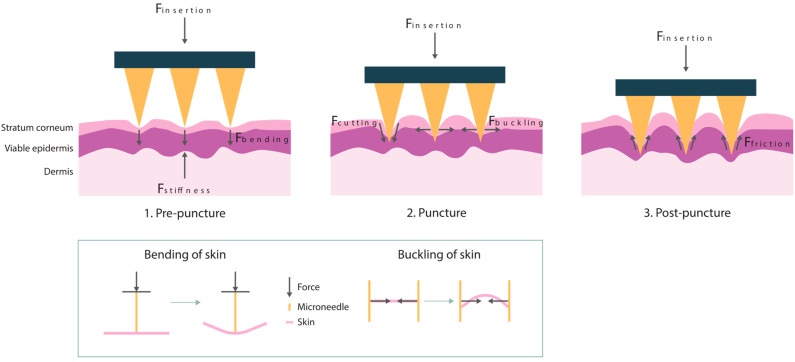
The insertion process of microneedles into the skin with the most important forces for each phase, pre-puncture, puncture, and post-puncture. A schematic representation of the effect of the bending and buckling force on the skin is shown below.

The insertion process of a DMNA is illustrated in Figure 1 and can be divided into three different phases. In the first phase, the pre-puncture phase, the skin will deform upon contact of the needle tip with the skin surface. In this phase, there are several resistance forces involved, namely the stiffness force (*F_stiffness_*), also referred to as indentation, and bending force (*F_bending_*). The stiffness force can be described as the vertical force provided by the skin during compression of the skin. The perpendicular loading of force by the needle on the skin causes the skin to bend [7]. Once maximal vertical deformation of the skin is achieved, puncturing of the skin occurs, which is defined as the second phase. The main forces during this phase are the cutting force (*F_cutting_*) and buckling force (*F_buckling_*). The cutting force is the force that is required to cut through the skin fibers. Simultaneously, the skin in between the needles must buckle to allow for this insertion to happen. This implies the compression of the skin by the pressure of the needle, which causes the skin to buckle [7,8]. Following the puncturing of the skin, the needles penetrate further into the skin during the post-puncture phase. In this phase, the friction force (*F_friction_*) mainly determines the penetration depth of the needles. This is a result of the rubbing of the needle surface against the skin tissue [7,9]. Lastly, the skin resistance force increases again if the backing layer of the DMNA touches the skin surfaces, which causes a physical constraint for further penetration of the DMNA [10]. The magnitude of these individual resistance forces is related to the dimensions and material of the microneedle, and the administration conditions like application speed.

**Figure 2:**
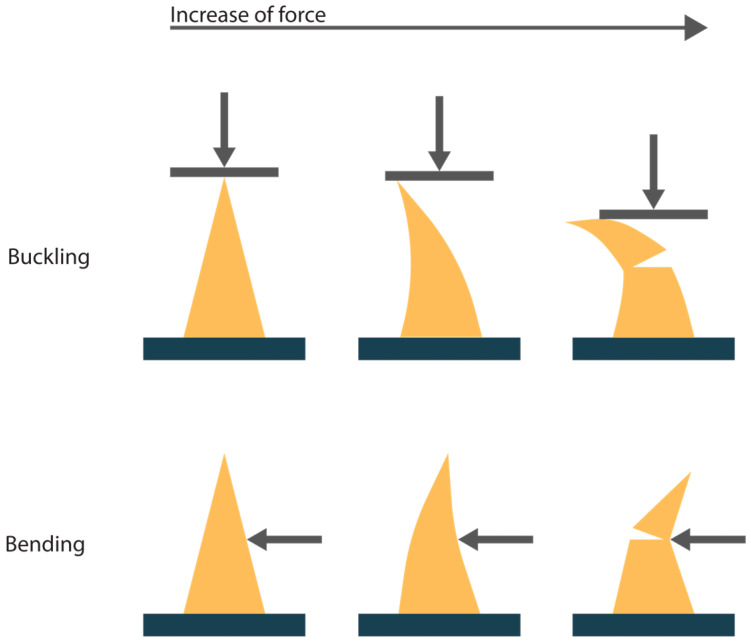
A schematic representation of the two fracture modes for microneedles upon application of force, buckling and bending. The arrow indicates the direction of the applied force.

If the insertion force is too small to overcome the skin resistance forces (Equation (1)), penetration will not occur. Another cause for unsuccessful penetration is fracture of the microneedle. In general, fracture modes are defined as buckling and bending of the needle tips, as illustrated in Figure 2. The needles can buckle due to the axial force that acts on the tips during the insertion. Bending of the needles can occur because of uneven skin surface or human error during the insertion [11,12]. The maximum buckling and bending force that the microneedle tips can withstand without signs of fracture can be described with Equations (2) and (3).

The maximum buckling force that a microneedle can withstand is defined by Euler’s formula for a column with one fixed and one free end. It is assumed that the slenderness ratio, which derives from the length and the least lateral dimension of a column, is greater than the critical slenderness ratio that determines the column to be ‘long’ [12,13,14]:

(2) $$F_{max\;buckling}=\frac{{C\pi }^{2}EI}{L^{2}}$$

where *C* is a constant with a value of 0.25 for columns with one fixed and one free end, *E* is Young’s modulus of the material, *I* is the area moment of inertia, and *L* is the length of a microneedle [12,15].

The maximum bending force that a microneedle can withstand is defined as follows [12,13]:

(3) $$F_{max\;bending}=\frac{\sigma_{y}I}{cL}$$

where *σ_y_* is the yield strength of the material, and *c* is the distance of the neutral axis to the outermost edge of the microneedle. Aggarwal et al. [13] define *c* as *d*/2, where *d* is the diameter of the circular base and as *a*/2, where *a* is the side of the square base of the column; *c* can be treated similarly for other shapes of the base. However, concerning a linearly tapered column instead of a straight one, *c* will vary with the length of the column. Therefore, it is assumed that *c* can be defined with *d_equivalent_* and *a_equivalent_* for circular and square-based tapered columns, respectively, as shown in Equation (4) [14].

(4) $${a\;or\;d}_{equivalent\;}={a\;or\;d}_{tip}+\frac{\left({a\;or\;d}_{base}-{a\;or\;d}_{tip}\right)}{3}$$

The area moment of inertia is defined as the resistance to bending due to the cross-sectional shape. Cross-sections with more material located further from the bend axis are more resistant to bending. This is reflected in an equation that depends on the cross-sectional shape. In general, for circular or square cross-sections, Equations (5) and (6) apply, respectively [12,14]:

(5) $$I_{circle}=\frac{\pi d^{4}}{64}$$

(6) $$I_{square}=\frac{a^{4}}{12}$$

where *d* is the diameter of the circular cross-section, and *a* is the side of the square cross-section. Again, these equations apply for straight columns, but the microneedles in this literature review are tapered columns. Therefore, we assume that the *d_equivalent_* and *a_equivalent_* (as defined in Equation (4)) can replace the diameter and side in Equations (5) and (6), respectively [14].

To ensure successful penetration, the safety index (Equation (7)) can be used to evaluate the DMNA’s performance. A safety index larger than one results in more reliable and stronger DMNAs [16].

(7) $$Safety\;index=\frac{F_{fracture}}{F_{insertion}}$$

Four requirements can be described for optimal drug delivery with DMNAs: (1) A high drug load to enable the delivery of larger doses of a drug, and therefore a high volume of the DMNA is required; (2) a high skin insertion ratio, which is defined as the number of punctuations in the skin divided by the total number of needles on the DMNA, and is required to deliver the loaded drug; (3) a high mechanical strength and toughness of the microneedle tip to avoid fracture and deformation of these tips; (4) a high penetration depth to improve the dissolution of the microneedle tip. By selecting the proper geometry of the DMNA, these requirements can be fulfilled. Regarding the mechanical strength of a microneedle, the material utilized as the matrix of the DMNA and the drug load is obviously of great importance. In general, a larger Young’s modulus (*E*) and yield strength (*σ_y_*) result in an increased fracture force, which can be deduced from Equations (2) and (3) [17]. However, the choice for the material of the matrix is highly dependent on the drug and desired release profile. Therefore, the different aspects related to the material choice were excluded from this review. This review will focus on the effect of the geometry of DMNAs on the aforementioned requirements, with the aim to answer the question ‘What is the optimal geometry of dissolving microneedle arrays?’.

## 2. Geometry

**Figure 3:**
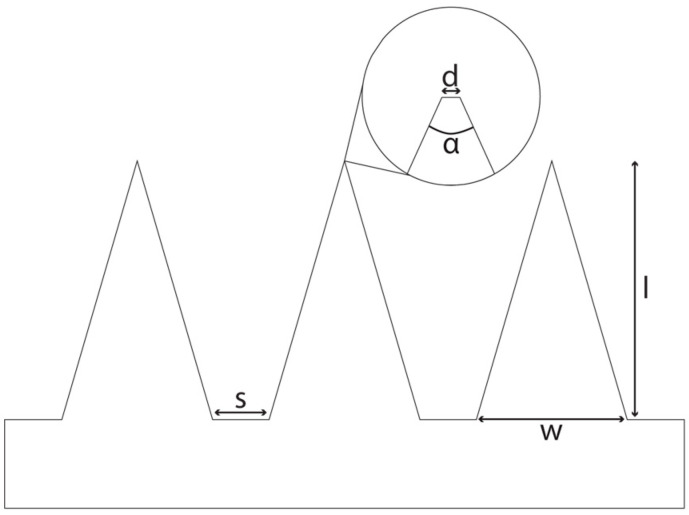
A schematical side view of a DMNA. s: spacing, base-to-base; d: tip diameter; α: tip angle; w: width of base; l: length.

As a starting point, a literature search was conducted in which various parameters with regard to the geometry of DMNAs were considered. All comparisons included in this review are from within one study to avoid inadequate comparison of more than one variable. We searched for the following parameters: shape, aspect ratio, length, width of the base, tip diameter and angle, and spacing. In Figure 3, a schematic overview is shown for the definition of the parameters.

### 2.1. Shape

**Figure 4:**
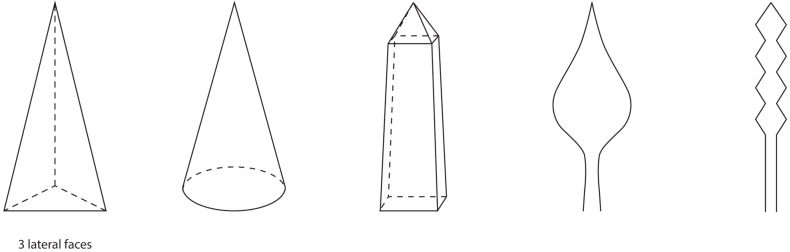
Shapes for microneedles, from **left** to **right**: triangular pyramidal, conical, obelisk, candle-flame, and barbed microneedle shapes.

Theoretically, there is an endless variety of possible shapes for the microneedle tips from which a part is shown in Figure 4. First of all, the method of manufacturing has a notable influence on the shapes that can be used. There are various methods available for the production of DMNAs, with the micro-mold technique being the most used one [18]. In this process, often a negative polydimethylsiloxane (PDMS) mold is made from a positive master array [4]. Subsequently, a solution is cast onto the PDMS mold followed by, for example, centrifugation to fill the needle cavities and remove entrapped air. Finally, a second solution, also referred to as the backing layer, may be applied after which the DMNA will be left for air drying [18]. This fabrication method limits the choice of shapes. Pyramidal, conical, or occasionally obelisk-shaped microneedles (Figure 4) have been prepared. However, more advanced shapes like candle-flame-shaped microneedles or backward-facing barbs on the needle (Figure 4) cannot be fabricated with PDMS molds [19,20,21,22]. Currently, the conical and pyramidal shapes are by far the most used shapes.

Regarding the pyramidal shape, a distinction can be made based on the number of lateral faces of a shape. Li et al. [23] and Loizidou et al. [24] fabricated microneedles with various numbers of lateral faces. First, the mechanical properties were presented as the compressed height of the microneedles after the application of 100 N for 5 s. Li et al. [23] did not observe a significant difference in the compressed height (Table 1). Next, the skin insertion ratio was assessed. Microneedles with six lateral faces showed a higher skin insertion ratio (76.2%) than those with four lateral faces (49.6%) [23]. In addition to these observations, during simulated buckling analyses, Loizidou et al. [24] found that with an increasing number of lateral faces, the microneedles were able to resist higher compressive loads, and thus are less likely to fail from buckling. A similar trend was experimentally found by Fitaihi et al. [25]. The results of Li et al. [23] and Loizidou et al. [24] imply that an increase in lateral faces is favored in the context of improving both the skin insertion ratio and mechanical properties.

Li et al. [23] also compared pyramidal microneedles with conical microneedles (Table 1). Although the mechanical properties were similar, the skin insertion ratio was higher for the conical than for the pyramidal microneedles (Table 1). This is in concordance with the observations of Tabriz et al. [16], who observed that the conical microneedles required less force to penetrate the skin than the pyramidal microneedles. The skin insertion ratio was not determined, but it was observed that the penetration depth in the skin of the conical microneedles was slightly higher than for the pyramidal microneedles. A similar effect or no difference was observed in the studies of Cordeiro et al. [26], Anbazhagan et al. [27], and Pünnel et al. [28]. The difference may be ascribed to a larger skin contact area after penetration, and therefore an increase in friction forces for the pyramidal microneedles compared to the conical microneedles [29]. The skin contact area after insertion is equal to the lateral surface area of the needle, which was more than 20% smaller for the conical needle than for the pyramidal needles [16,26]. However, these friction forces occur once the microneedle is inserted, and in the study of Li et al. [23], a difference in skin insertion ratio was also observed. The lateral surface-to-volume ratio may explain this difference, because with a lower lateral surface-to-volume ratio, the skin insertion ratio increases (Table 1). The attribution of the lateral surface-to-volume ratio to the skin insertion ratio is related to the application force because the lower the lateral surface-to-volume ratio, the smaller the distribution area of the force. Therefore, it is hypothesized that the insertion force on the area between the tip of the needle and the skin is larger for the shapes with relatively lower lateral surface-to-volume ratios, which will result in higher skin insertion ratios. Tabriz et al. [16] also observed that a larger insertion force was required for pyramidal microneedles with four lateral faces than for conical microneedles, which supports this theory.

Apart from the skin insertion ratio, the mechanical properties were not significantly different between the conical and pyramidal needles [23]. Fitaihi et al. [25] found that the conical shape is more resistant to a compressive load than the pyramidal shape with three or four lateral faces. However, Tabriz et al. [16] observed that the fracture force increased in the following order: pyramidal with four lateral faces > conical > pyramidal with three lateral faces. This may be associated to the change in the surface area of the base. A decrease in the base surface area will result in a decreased cross-sectional area to support the tip during the mechanical test [30].

In conclusion, the most favorable needle shape has a small lateral surface area to minimize the friction forces and thereby maximize the penetration depth. Furthermore, it should have a small lateral surface-to-volume ratio to maximize the insertion force on the tip of the microneedle. These two characteristics can be found in a conical microneedle.

### 2.2. Aspect Ratio

The aspect ratio is the length-to-width of the base ratio. In general, an increase in the aspect ratio of the microneedle results in a decrease in the mechanical strength of the needle tips (Table 2) [16]. This reduction in the mechanical strength of the microneedle results from the reduction in maximum buckling and bending force that a microneedle can withstand, Equations (2) and (3), respectively, because of either a longer needle (*L*) or a smaller area moment of inertia (*I*) with an increase in the aspect ratio. Consequently, the safety index, Equation (7), can become near or below one, which implies a high risk of microneedle fracture upon insertion. On the other hand, an increase in aspect ratio can increase the skin insertion ratio (Table 2). However, when Lim et al. [31] increased the aspect ratio from 2 to 3, a decrease in the skin insertion ratio from 80% to 60% was observed. This observation may be explained by needle fracture due to the reduced mechanical strength with the increase in the aspect ratio.

A high aspect ratio is preferred to achieve a maximal skin insertion ratio. However, the mechanical strength decreases with an increasing aspect ratio. An aspect ratio between 2 and 4 is considered to provide the optimum balance for these conflicting requirements.

### 2.3. Length

Initially, the assumption may be that a longer needle improves the drug delivery efficiency because of an enlarged drug loading capacity and increased penetration depth. However, the latter also implies an increase in the pain experienced by the recipient. Nonetheless, the associated pain is still substantially less than for using hypodermic needles [36,38]. Furthermore, the micro-molding process should be taken into account, because Chu et al. [32], for example, showed that it is difficult to remove microneedles longer than 1 mm from a PDMS mold.

For a successful penetration, a minimal length of the tip is required to overcome the tissue compression resulting from the elastic nature of the skin [39]. Apart from the length of the needle, factors other than geometry influence the skin insertion ratio and penetration depth as well. A higher application speed may prevent the wrapping of the skin around the needle, because of the elastic properties of the skin. Upon deformation, the skin will return to its original state. If the insertion time, determined by the speed of application, is shorter than the relaxation time of the skin, the elasticity of the skin plays a less prominent role during microneedle application [40,41]. Apart from the application speed, the penetration depth is also dependent on the application force. Obviously, an increase in application force results in an increased penetration depth [34,42]. In pre-clinical experiments, the choice of the ex vivo model is also of importance. The skin thickness of the used ex vivo model will determine the degree of tissue compaction upon insertion of the microneedles. This will affect the minimum required length to overcome the elastic compression of the skin for penetration [43,44]. Therefore, the varying experimental conditions in different studies make it impossible to compare results originating from different studies. The results presented in Table 2 are, therefore, only compared within the respective study and not between the different studies.

The penetration depth depends on the degree of skin deformation. The bending and buckling of the skin prevent the needles from penetrating further, because skin in between the needles hits the backing of the DMNA. The impact of this issue can be reduced through increasing the needle length (Table 2) [32]. However, it is important to note that, regardless of the needle length, 10% to 20% of the original needle length does not penetrate the skin because the skin, between the needles, hits the backing of the DMNA [34]. In addition, Li et al. [36] observed that there was no linear proportional relationship between an increase in length and increase in penetration depth for needles with a length between 800 and 1500 µm [16,36]. This may explain the observed relative higher drug delivery efficiency for shorter microneedles [36]. It implies that despite a higher possible drug load of longer needles, the relative loss of drug will be higher than for the shorter needle because of the relatively lower penetration depth of the longer needle.

Next to the penetration depth, it is also essential to consider the mechanical strength of the microneedle. In general, the mechanical strength decreases with increasing microneedle length at equal widths of the base (Table 2) [17,31,45]. This is also in concordance with a decrease in the maximum buckling and bending forces of the needles, for an increase in length (Equations (2) and (3), respectively).

As mentioned above, the application speed and force are of main importance for a successful penetration. Hypothesizing a relatively low application speed and force, it is thought that a minimum length of 400 µm will result in successful penetration. Furthermore, an increase in length will provide a higher drug loading capacity because of the larger volume of the microneedle, and is therefore favorable. However, increasing the length, while keeping the width of the base equal, results in a reduction in the mechanical strength of the needle. Therefore, a compromise should be made between the maximal drug loading capacity and required mechanical strength. We estimate that a microneedle length between 600 and 1000 µm can fulfill these requirements.

### 2.4. Width of Base

The width of the base will ensue from the length and aspect ratio. As mentioned earlier for the aspect ratio, a decrease in the width of the microneedle increases the skin insertion ratio but decreases the mechanical strength of the microneedle [17,45,46]. This can also be derived from Equations (2) and (3) for the maximum buckling and bending forces, respectively, because of a lower area moment of inertia (*I*).

### 2.5. Tip Diameter and Angle

Regarding the tip of a microneedle, there are two parameters to consider: tip diameter and angle. The tip diameter determines the interfacial area, which is the area that is in first contact with the skin upon insertion. The angle of a tip is determined by the aspect ratio of the microneedle. Both tip diameter and angle are of main importance at the start of the insertion process. In several studies, it was shown that the insertion force increases proportionally with an increase in interfacial area. The insertion force was determined as the drop in applied force or skin resistance [46,47,48,49]. The relationship of the interfacial area and insertion force can also be deduced from Equation (8), which can be substituted into Equation (1) [11]:

(8) $$F_{stiffness}=P_{pierce}\times A$$

where *P_pierce_* is the pressure that is required to pierce the microneedle into the skin, and *A* is the tip surface area.

In accordance with a larger required insertion force for larger interfacial areas, Lim et al. [31] observed a significantly lower skin insertion ratio for microneedles with a tip diameter of 200 µm than for those of 50 or 100 µm with equal application forces. Römgens et al. [49] evaluated the influence of the microneedle tip diameter on the penetration depth. Initially, the skin is more compressed instead of penetrated for a larger tip diameter. Therefore, the penetration depth was significant larger for a tip diameter of 5 µm than for those of 24 and 37 µm at low application forces. With increasing the application force, there was no significant difference in penetration depth for the various tip diameters [49]. He et al. [50] studied the effect of the tip radius, in a range of 5 to 22 µm, on the piercing force. It was demonstrated that the smaller the tip radius, the smaller the piercing force.

Despite the benefits of a smaller interfacial area, it can be derived from Equations (2) and (3) that a smaller interfacial area results in a reduction in the mechanical strength of the microneedle because of the smaller area moment of inertia (*I*). He et al. [50] presented that the calculated buckling force for a tip radius of 5 µm was smaller than the observed piercing force. However, the fracture force was not experimentally confirmed, and penetration was observed for a tip radius of 5 µm [50]. Lim et al. [31] did not observe a significant difference in the number of intact microneedles after the application of 40 N when tip diameters where varied from 50 µm to 200 µm, indicating that within this tip diameter range, the mechanical properties were sufficient to ensure efficient skin penetration without fracture. However, it is unknown whether the mechanical strength of the microneedles with a tip diameter smaller than 50 µm will be unacceptably reduced. On the other hand, a reduced mechanical strength, due to a smaller tip area, is not by definition a problem because the insertion force will also be reduced (Equations (1) and (8)) [51].

Furthermore, the manufacturing method of the microneedle master array may determine the tip diameter, as limitations in resolution of the manufacturing process can restrict the extent to which the tip diameter can be reduced.

Economidou et al. [29] compared various tip angles (Figure 3). The insertion force for the microneedles with a tip angle of 97° was greater than for those of 48.5°. Such a trend was also observed by Aoyagi et al. [52], Liu et al. [53], and Ahn [54]. The larger interfacial area upon and during insertion of the microneedles can explain this trend of increasing insertion forces with increasing tip angles [53]. As shown in Equation (8), a larger interfacial area results in a larger stiffness force and consequently a larger insertion force (Equation (1)).

In conclusion, the tip diameter should be adjusted to the smallest tip diameter feasible, but this depends on the manufacturing method of the microneedle master array and the material of choice for the preparation of the DMNA. Preferably, the tip angle is the smallest possible, but this parameter is determined by the aspect ratio mentioned earlier.

### 2.6. Spacing

As presented in Equation (1), the insertion force depends on, amongst other factors, the bending and buckling forces of the skin. The spacing, which is the distance between two adjacent needles (Figure 3), affects these forces [7]. In general, the narrower the spacing, the larger the required insertion force per patch due to the ‘bed of nails’ effect. This effect appears because of the reduced force per needle tip with increasing needle density and equal patch surface area, so an increase in the number of needles [34]. For the ‘bed of nails’ effect, the patch surface area is equal and the density of needles varies, so the total number of needles is higher with a decrease in spacing. However, if the total number of needles is equal and the spacing increases, an increase in the skin insertion ratio and decrease in the required insertion force is observed.

**Figure 5:**
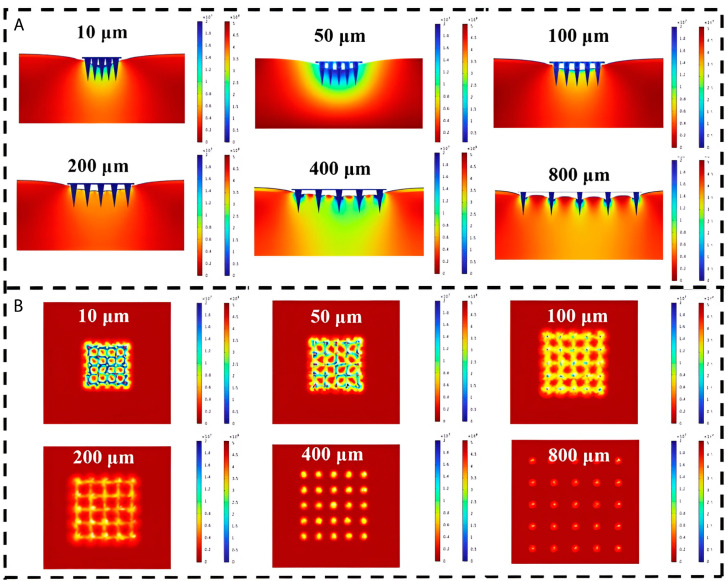
Cross section (**A**) and top view (**B**) of stress distribution cloud plot against interval spacing. Reproduced and adapted with permission from Liu et al. [53].

During needle insertion, the stress fields in the skin per needle will overlap for smaller spacings (Figure 5). Consequently, a larger insertion force is required because of the relative increase in stiffness force (Equation (1)). From a certain interspacing, the stress fields are isolated, and increasing the spacing further will not result in smaller insertion forces anymore [7,10,41,53]. This was shown in several studies in which cut-off values for spacing varying from 150 to 200 µm were found for microneedles with a length between 600 and 700 µm [7,10,53]. Concerning these overlapping stress fields, it appeared that an increase in the length of the microneedle contributes to a larger size of the stress field. Li et al. [36] investigated the effect of the length of the microneedle and the spacing on the delivered dose. It was observed that the dose delivery efficiency increased from 20% to 60% when the spacing was increased from 400 to 850 µm for needles with a length of 1000 µm. For the needles with a length of 800 µm, the dose delivery efficiency was 60% for both spacing distances.

In the decision for the dimension of the spacing, a compromise should be made between a large spacing to reduce the insertion force and enhance the skin insertion ratio and a small spacing to maximize the drug loading per patch. According to the abovementioned cut-off values of 150 to 200 µm for needles with a length between 600 and 700 µm and that an increase in length requires a higher cut-off value, a spacing of 300 µm would be a reasonable starting point.

## 3. Conclusions

To answer the question ‘What is the optimal geometry for dissolving microneedles?’, several parameters have been taken into consideration in this literature review. For the highest drug delivery efficiency, the skin insertion ratio, penetration depth and the avoidance of fracture or deformation of the DMNAs were discussed for the different parameters. First of all, it was found that with increasing the number of lateral faces, the lateral surface-to-volume ratio decreases, but it is at its lowest for a conical shape and, therefore, appears to be the most optimal shape for a microneedle. This reduction in lateral surface-to-volume ratio results in a larger applied force on the needle tip, which is in first contact with the skin upon insertion and, therefore, the required insertion force is reduced. Regarding the needle tip area, it is best to keep this as small as possible to reduce the required insertion force. Evolving from this, the tip diameter and tip angle should be as small as possible. The maximal reduction of the tip diameter is often limited by the manufacturing method of the master array as well as the properties of the materials used for the DMNA. In addition, the tip angle is determined by the aspect ratio and the length of the needle. In general, it can be concluded that a high aspect ratio results in an improved skin insertion ratio compared to microneedles with a low aspect ratio. However, with increasing the aspect ratio, there is also an increased risk of fracture due to a reduced maximum buckling and bending force that a microneedle can withstand. Therefore, the minimum safety index of one can be utilized to achieve a DMNA with the highest aspect ratio while avoiding fracture. Lastly, the spacing of the microneedles has to be taken into consideration. A small spacing will result in the highest drug loading efficiency, but it was found that the skin insertion ratio was decreased and the insertion force was increased for a small spacing in comparison to a large spacing because of overlapping stress fields of the individual needles in the skin. Taking all of these aspects into consideration, it can be concluded that it is impossible to provide fixed values for the various parameters concerning the geometry of microneedles. However, as a starting point, we recommend choosing a conical shape for the microneedles with a length between 600 and 1000 µm, an aspect ratio between 2 and 4, and a minimum spacing of 300 µm.

## Figures and Tables

**Table 1:** Characteristics of conical microneedles and pyramidal microneedles with six or four lateral faces [23]. Equations used to calculate the lateral surface area and volume can be found in Appendix A.

Microneedle Shape	Lateral Surface Area in % of Conical	Volume in % of Conical	Lateral Surface-to-Volume Ratio	Skin Insertion Ratio in %	Compressed Height in % ^1^
Conical	100	100	1.0	97.8	77.0
Pyramidal with six lateral faces	72	54	1.3	76.2	78.3
Pyramidal with four lateral faces	68	42	1.6	49.6	76.3

^1^ Compressed height is the height after pressure and is a parameter for the mechanical properties.

**Table 2:** Overview of the properties of the microneedles depending on their length and/or aspect ratio, which is length divided by width of the base (experimental conditions vary per study).

	Length in µm and (Aspect Ratio)	Mechanical Strength in % of MN Intact	Skin Insertion Ratio in %	Penetration Depth in µm
Chu et al. [32]	600 (2)	-	-	-
	900 (3)	-	-	-
Donnelly et al. 2011 [33]	300 (1)	94	0	-
	600 (2)	92	87	-
	900 (3)	95	100	-
Donnelly et al. 2010 [34]	350 (1.2)	-	-	293
	600 (2)	-	-	470
	900 (3)	-	-	789
Kochhar et al. [35]	957 (6.1)	-	73	-
	1336 (5.7)	-	52	-
Li et al. [36]	800 (4)	-	96	704
	1000 (5)	-	90	560
	1200 (6)	-	91	684
	1500 (7.5)	-	90	765
Lim et al. [31]	400 (1)	~90	~50	-
	800 (2)	~80	~80	-
	1200 (3)	~60	~60	-
Peng et al. [37]	634 (2)	-	29	252
	970 (3)	-	88	504

## Appendix A

(A1) $$l_{cone}=\sqrt{h^{2}+r^{2}}$$

(A2) $${LA}_{cone}=\pi \cdot r\cdot l_{cone}$$

(A3) $$l_{pyramid}=\sqrt{h^{2}+a^{2}/2}$$

(A4) $${LA}_{rectangular\;pyramid}=2\cdot a\cdot l_{pyramid}$$

(A5) $${LA}_{hexagonal\;pyramid}=3\cdot a\cdot l_{pyramid}$$

where *l* is the slant height, *h* is the height, *r* is the base radius, *a* is base side length, and *LA* is the lateral surface area. Assumption: all sides of the pyramidal are equal.

(A6) $$V_{hexagonal\;pyramid}=\frac{1}{3}\times BA\times h$$

(A7) $${BA}_{cone}=\pi \times r^{2}$$

(A8) $${BA}_{rectangular\;pyramid}=a^{2}$$

(A9) $${BA}_{hexagonal\;pyramid}=\frac{3\times \sqrt{3}{\times a}^{2}}{2}$$

where *V* is volume and *BA* is base surface area.
